# “If he has education, there will not be any problem”: Factors affecting access to education for children with disabilities in Tamil Nadu, India

**DOI:** 10.1371/journal.pone.0290016

**Published:** 2023-08-16

**Authors:** Kumudha Aruldas, Lena Morgon Banks, Guru Nagarajan, Reeba Roshan, Jabaselvi Johnson, David Musendo, Isaac Arpudharangam, Judd L. Walson, Tom Shakespeare, Sitara S. R. Ajjampur

**Affiliations:** 1 The Wellcome Trust Research Laboratory, Division of Gastrointestinal Sciences, Christian Medical College, Vellore, Tamil Nadu, India; 2 International Centre for Evidence in Disability, London School of Hygiene & Tropical Medicine, London, United Kingdom; 3 Department of Physical Medicine and Rehabilitation, Christian Medical College, Vellore, Tamil Nadu, India; 4 Department of Developmental Paediatrics, Christian Medical College, Vellore, Tamil Nadu, India; 5 Departments of Global Health, Medicine, Pediatrics, and Epidemiology, University of Washington, Seattle, Washington, United States of America; 6 The DeWorm3 Project, University of Washington, Seattle, Washington, United States of America; University of Hyderabad, INDIA

## Abstract

This study explores factors affecting children with disabilities’ enrolment and experience in school in Tamil Nadu, India. In-depth interviews were conducted with 40 caregivers and 20 children with disabilities. Children were purposively selected to maximise heterogeneity by gender, impairment type and enrolment status, using data from a previous survey. Overall, caregivers recognised the importance of school for their children’s future livelihoods or at least as a means of socialisation. However, some questioned the value of school, particularly for children with intellectual or sensory impairments. Other barriers to school enrolment and regular attendance included poor availability and affordability of transport, safety concerns or school staffs’ concerns about children’s behaviour being disruptive. While in school, many children’s learning was limited by the lack of teacher training and resources for inclusive education. Poor physical accessibility of schools, as well as negative or overly protective attitudes from teachers and peers, often limited children’s social inclusion while in school. These findings carry implications for the implementation of inclusive education in India and elsewhere, as they indicate that despite legislative progress, significant gaps in attendance, learning and social inclusion remain for children with disabilities, which may not be captured in traditional metrics on education access.

## Introduction

Childhood education is linked to many benefits to individuals, their households, communities, and countries, including increased future earnings, improved health outcomes, and overall well-being, as well as improved socio-economic development and decreased inequalities [1]. The importance of equitable access to quality, inclusive education for all children is recognised in many national and international laws and frameworks, including the Sustainable Development Goals 4 [2, 3]. However, globally, around 240 million children with disabilities frequently remain excluded from the benefits of education [4]. The right of children with disabilities to education is recognized explicitly by 37 national constitutions and in other laws and policies in 113 countries [3]. Article 24 of the United Nations Convention on the Rights of Persons with Disabilities (UNCRPD), which has been ratified by 185 countries, also codifies the rights of children with disabilities to accessing education without discrimination [5].

Despite these laws and policies, children with disabilities are less likely to attend school, progress to secondary and higher levels of education, and have lower overall attainment compared to their peers without disabilities, particularly in low-income countries (LMICs) [4, 6]. Across 15 LMICs, children with disabilities were, on average, 31 percent less likely to attend school then non-disabled peers [6]. Children with intellectual and communication impairments are particularly likely to be excluded from school [7]. Even when children with disabilities do attend school, skill acquisition is often hindered by the lack of provisions for an inclusive education, including inadequate teacher training, inaccessible facilities, and poor availability of specialist education resources [8, 9]. Further, children with disabilities frequently have negative social experiences while at school due to isolation, discrimination and violence from teachers and peers [10, 11].

India is an important country to assess and impact the access of children to an inclusive education, given its population size, policy climate, and regional and global influence. The 2011 Census estimated that there are at least 65.8 million children of school-going age (5–19 years) who have a disability [12]. This number is recognized to be underestimated, given the restrictive definition of disability used in the Census [13]. The Constitution of India guarantees the right of all children to education and Government policies in India have increasingly focused on improving education for children with disabilities [14, 15]. The Right of Children to Free and Compulsory Education Act (RTE) 2009 builds on the rights in the Constitution, and explicitly codifies the rights of children with disabilities to primary education, and prohibits discrimination based on disability [16]. The Rights of Persons with Disabilities Act 2016 defines inclusive education as a “system of education wherein students with and without disabilities learn together and the system of teaching and learning is suitably adapted to meet the learning needs of different types of students with disabilities” [17]. This Act outlines active identification of disabilities amongst children in school, making facilities accessible, providing specialist educational resources, and other modifications for children aged 6–18 years having 40% or more difficulty and are attending school at a government-funded or recognised institution.

The National Policy on Education 2020 also includes a significant focus on improving access to and quality of education for children with disabilities. For example, it calls for mainstreaming disability-inclusive teaching methods into standard teacher training [18]. Specialist educators, particularly for middle and secondary schools, were also recognized as a priority area for investment, as were resource schools that can provide specialist educational resources and teaching strategies for children with multiple or severe disabilities. Further, the Government of India supports two national programs, Sarv Shiksha Abhiyan (SSA) and the Rashtriya Madhyamik Shikhsha Abhiyaan, for promoting access to and quality of primary and secondary education respectively for all children, including children with disabilities [19]. They include specific provisions for children with disabilities, such as specialist curricula and teachers, access to assistive devices and other learning supports, and medical and educational assessment. While far ranging in scope, in practice there have been large gaps in their implementation [1]. For example, official data indicates an increase in the number of schools that have been upgraded for accessibility, but many have experienced faulty provision (e.g., installation of non-useable ramps) [20].

There has been an evolution in India’s policies towards inclusive education. Colonial and early post-colonial approaches to disability and inclusion, including in education, have focused predominantly on a policy of segregation [21]. In 1966, the Kothari Commission recognised the importance of including children with disabilities in the Indian education system [22]. It set up a dual approach that has been the dominant model of education for children with disabilities in India, in which there is an emphasis on both encouraging enrolment of some children with disabilities into mainstream schools while also focusing on the creation of special schools. More recently, there has been more of a shift to “inclusive education”, such as in the 2016 Rights of Persons with Disabilities Act and the 2016 National Education Policy, although segregated schools are still common [19].

There is some evidence that the enrolment of children with disabilities in India has increased in the past decade. For example, the proportion of children with disabilities in primary schools increased by 50% between 2011 and 2016, from 0.8% to 1.2% of all children enrolled [19]. However, the proportion of children with disabilities in secondary school has remained unchanged (around 0.3%) over this time.

Further, these figures only capture children with official certifications of disability and do not provide information on the proportion of children with disabilities out of school. Even with increased enrolment, studies have highlighted persistent barriers to accessing and benefitting from education in different regions of India. These barriers include institutional challenges, including inadequate teacher training, inaccessible facilities, insufficient adaptations, and specialist resources, and negative attitudes toward disability held by teachers and peers without disabilities [8, 23]. Other barriers at the community and household-level include poverty, lack of accessible and affordable transport, and caregiver attitudes about the ability of children with disabilities to learn and the value of education [8, 24]. Additional research is needed to explore the experiences of children with disabilities in accessing education in different contexts, including barriers and facilitators. Consequently, this study focuses on the perspectives of children with disabilities and their families and caregivers in Tamil Nadu, India to understand the factors affecting decisions on if and where to enrol, how children got to school, their social and academic experience while at school, and learning outside the classroom.

## Materials and methods

### Study area and setting

This study was carried out in two sites, a rural block in Ranipet district, Timiri (formerly Vellore district) and a tribal block in Tiruvannamalai district, Jawadhu Hills, both in Tamil Nadu in southern India. These communities were chosen as this research was linked to an ongoing study in these areas with censused populations (DeWorm3 study) [25]. The ongoing DeWorm3 study is a multi-country, cluster-randomised controlled trial to test the feasibility of interrupting transmission of soil-transmitted helminths using community-wide mass drug administration in all ages compared to the standard of care (targeted deworming including pre-school and school aged children) [25]. As part of the DeWorm3 study, a baseline census (in 2017) of all individuals residing in the two study sites enumerated a population of 140,932 individuals [26]. During the DeWorm3 census update in 2019–20, children in the ages 5–17 years were screened for disability using the UNICEF/Washington Module on Child Functioning [27]. This module uses caregiver-reporting to identify children with functional limitations across a range of activities (e.g., in seeing, hearing, walking, communicating, and cognition). For most activities, functioning is reported on a scale of “no difficulty”, “some difficulty”, “a lot of difficulty” or “cannot do”. In line with international standards, disability was defined as experiencing “a lot of difficulty” or “cannot do” on at least one domain [28]. The census found a prevalence of childhood disability (5–18 years) of 1.0% in this population with significantly fewer children with disabilities enrolled in school compared to those without disabilities (57% vs 90%). Non-attendance of children with disabilities was more common among older children, children living in poorer households, or those who lived in Jawadhu Hills, a tribal community.

### Study design and recruitment

In-depth interviews were conducted with caregivers and children with disabilities between the ages of 5–17 years from January–July 2020. The children were purposively selected from amongst the 299 children identified as having a disability during DeWorm3 project census to maximise heterogeneity by gender, age, and type and severity of functional limitations (“a lot of difficulty” or “cannot do”), and to have an equal balance of children in and out of school. Forty primary caregivers of children with disabilities were interviewed about their child’s experience in school. They were approached by a member of the research team and if they agreed, an appointment was made for an interview at their home. Further, 20 of these children with disabilities (10 in school and 10 out of school) were selected to be interviewed directly. The primary caregivers were almost all family members, particularly mothers. Children selected to be interviewed directly were primarily older and able to communicate with available supports.

### Data collection and analysis

Semi-structured interview guides were developed for caregiver and child interviews. The caregiver interview guides focused on, 1) family background; 2) child’s disability, health, and access to disability-related support services; and 3) child’s education, including reasons for non-attendance and/or their social and learning experience while at school. The children’s interviews focused on the child’s “school journey”, exploring their experiences at home, getting to school, in the classroom, in the playground, and in using school facilities. Visual aids were used to prompt discussions, including emotion cards (faces with different expressions, such as “happy”, “sad”, and “angry”). Four interviewers received extensive training in the study protocols and disability. Interviews were conducted in Tamil, the local language, by experienced qualitative interviewers along with notetakers. All interviews were audio-recorded, transcribed verbatim, and translated into English.

A codebook was developed using *a priori* codes based on the interview guides. A small number of additional codes were added inductively based on emerging findings. Each transcript was coded independently by two researchers (one from each institution) using the codebook on ATLAS.ti 9.2 software. Coders had experience working on disability and education, and/or on the context of Tamil Nadu. Coding decisions, including differences between coders, were discussed by the research team by video conferencing or over email and with a third researcher when consensus was not reached between primary coders. Except for authors involved in data collection, others did not have access to participants identifiable information. The mapping of codes into themes was decided jointly by the two research teams. The interrelationships between themes and different participant characteristics (e.g., child’s gender, impairment type) were also examined.

### Ethics statement

Ethical approval for this research was received from the Human Subjects Division at the University of Washington (STUDY00000180) and the Institutional Review Board at Christian Medical College, Vellore as an amendment to the DeWorm3 study in India (10392 [INTERVEN]; IRB–A08, December 28, 2019). Informed written consent was obtained from all caregivers who were interviewed. Caregivers’ consent and child assent—verbal for interviews with children 7–11 years and written for children 12–17 years, were obtained for interviewing. Adaptations were available to support the direct participation of children with disabilities, including sign language interpretation and simplified information sheets/topic guides.

## Results

In total, information about the schooling of 40 children with disabilities was gathered through 40 caregivers and 20 child interviews (Table 1). All the selected children or caregivers agreed to participate. Of these 40 children included in the final sample, 20 (10 boys and 10 girls) were enrolled in school and 20 (10 boys and 10 girls) were not going to school at the time data was collected. All the 40 children selected for the study had varying degree of functional limitation ranging from ‘a lot of difficulty’ to ‘cannot’ in mobility, cognitive, vision, hearing, and anxiety/depression. Except for four children living with their aunts or grandparents, all were living with their biological parents. They mostly belonged to families engaged in farming as daily labourers on others’ land and a few belonged to families engaged in skilled occupations such as masonry, weaving, and hairdressing. A little over half the children (23 out of 40) lived in a nuclear family and the rest were in extended families. The experience of families sending their child to school and of children going to school is presented under four key themes, (i) enrolment and attendance, (ii) getting to school (iii) experience at school, and (iv) learning outside of school.

**Table 1:** Description of the study sample.

Characteristics		Number
Caregivers interviewed (40)	of girls in school	10
	of girls out of school	10
	of boys in school	10
	of boys out of school	10
Children interviewed (20)	Girls in school	5
	Girls out of school	5
	Boys in school	5
	Boys out of school	5
Type of disability (40)	Vision	4
	Hearing	3
	Mobility	19
	Cognition	8
	Mental Health	6
Caregiver-child relationship (40)	Mother	27
	Father	7
	Aunt	3
	Grandmother	2
	Sister	1
Level of schooling among enrolled children (20)	Special school	1
	Primary education (Grade 1–5)	2
	Middle education (Grade 6–8)	8
	Secondary education (Grade 9–10)	5
	Higher secondary education (Grade 11–12)	4
Level of schooling among out of school children (20)	Never went to school	2
	Special school	1
	Pre-school	1
	Primary education (Grade 1–5)	6
	Middle education (Grade 6–8)	5
	Secondary education (Grade 9–10)	4
	Higher secondary education (Grade 11–12)	1

### Enrolment and attendance

#### Attitudes on education

In general, the child’s parents jointly decided on whether or not to send their child to school, even in an extended family household. A common reason for sending children to school was that many considered education essential to get jobs and therefore, a better life. Some parents felt that their child with a disability would not be able to perform jobs that were prevalent in their area, such as jobs relying heavily on manual labour, and so considered education important for gaining access to desk-based jobs.

*“We are educating him because he must not be idle and stay in the house without knowing anything…*. *We provide education for the sake of education…*. *Currently*, *many are working from home on the computer; likewise*, *he can also do that work through education; without education*, *he cannot work*. *Education might have an important role; if he has education*, *there will not be any problem*.*”*

(Father of a boy, mobility limitation–“cannot do”)

Some parents, particularly parents of children with intellectual disabilities, reported that schooling could also help with their child’s overall development. Some acknowledged that their child may not be gaining the same academic skills as other children, such as literacy or numeracy, but they nonetheless felt it important for them to be with their peers for socialisation:

*“Everyone in the family [decided to send her to school]*. *Only if she studies*, *there will be brain development*. *Let her go*, *until she wants to go*. *That’s why we send her*. *If she is inside the house her mind will get affected so*, *if she is going to school*, *she will have some clear thoughts*.*”*

(Mother of a girl, cognitive limitation- “a lot of difficulty”)

Few children (8 out of 38) had ever been to a school had started school late between the ages of 6–8 years, however, the commitment of some parents to sending their child to school was evidenced by the personal efforts they took to ensure their child attended. Several parents spent significant time and effort dropping and picking up their child at school and sometimes also visit their child in school during the lunch break. For example, the father of a child having a lot of difficulty with mobility stayed at school throughout the day to provide assistance to his child. Almost all children in school indicated that they liked going to school. However, few of the children out of school reported not liking school, often linked to instances of teasing or abuse when previously enrolled.

The main reasons reported for why children were not in school were complex and often involved multiple concerns or barriers. Cited reasons included poor availability and affordability of transport, fear for the child’s safety and adequate management of their impairment (e.g., falling down due to mobility impairment or seizures with the embarrassment of getting clothes dirty or soiled), the child being disruptive in class or getting teased or abused by other children. Some caregivers questioned the value of school, particularly for children with intellectual impairments or profound sensory impairments, and if their child would be learning anything in school.

*“They did not teach him [went to school till 7th standard]*. *They can teach him only when he can speak; he cannot speak*, *so he went*, *sat idly and came back we cannot teach him*, *and he cannot even talk*.*”*

(Mother of boy, cognitive limitation–“a lot of difficulty”)

Gender could also intersect with disability as a factor hindering school enrolment. Menstrual hygiene management was an additional concern for girls after puberty and caregivers encouraged them to just stay at home while menstruating. Importantly, attaining puberty was also a reason for a parent to discontinue sending their daughters to school. Teachers’ attitudes could also affect decisions to send children to school. In three instances, the teachers had advised the parents not to send their children to school. In these instances, teachers stated that the child was or was perceived to be restless and disruptive in class.

#### Choice of school

Among the 38 children who were currently or ever enrolled in school, 32 children attended regular government schools. These were primarily co-education schools, where girls and boys studied together. The cost of sending a child to a government or a private school was a major determinant in the choice of school. There is no school fee in government schools, and the children receive free books and uniforms until class 10. Still, most caregivers reported miscellaneous expenditure for school bags, stationery, and replacing lost books and notebooks. Most caregivers reported that they would prefer sending their children to a private school, as they were perceived to offer a better-quality education. Yet only two children were going to private schools, where caregivers reported that their children were doing well in school. The costs associated with private schools, however, were difficult for many families to meet.

*“My husband told me that we will not be able to pay the fees [for a private school]*, *so we will admit him in government school*. *I said we have only two children*, *we have to educate them well… but since my husband passed away I am not able to pay the school fees [private school]…*. *Right now*, *I am thinking of stopping him from this [private] school*. *I will think about a school*, *where I do not have to pay so much*.*”*

(Mother of a boy, vision- “a lot of difficulty”)

Only two children had ever attended a special school for children with disabilities. One child with cognitive impairment attended a special school mainly to receive physiotherapy and because it was felt he would be disruptive to others in a government school. Some caregivers had been advised to send their child to a residential special school, however, they did not want to be separated from their child. They also perceived that care at residential special schools would be inadequate. For example, the caregiver of a child with a cognitive impairment explained the negative experience they had in sending their child to a special school. These negative experiences ultimately led them to withdraw their daughter from the special school and enrol her in a government school, even though they felt her education may have been better in this setting.

*“It [residential special school] is a hostel for children with poor brain development*. *That place was full of flies*. *Then*, *a week later they shaved her head*, *without asking us… She cried when she saw us… if she was there in the hostel*, *she would have got some knowledge*.*”*

(Aunt of a girl, cognitive limitation- “a lot of difficulty”)

#### Regularity of going to school

Most enrolled children attended school regularly except when they had an occasional illness such as fever or cold. However, over a third reported being absent from school two more days in a week for reasons like not having anyone available to drop them at school, lack of available transportation, worsening severity of the disability and underlying health conditions, and fear of getting scolded by the teachers. For example, the mother of a girl with progressive mobility impairment described that her daughter often refuses to go to school even though the children and teachers at school were friendly to her as she is worried about falling over and hurting herself:

*“… She is been weaker in this one year; it [disability] increased after giving her tablets bought from Madras … It [going to school] depends on her mood…*. *She is falling and getting injured… if she wishes to go to school*, *she will get ready and go*. *Otherwise*, *even if we make her get ready and drag her out of the house and beat her up*, *she will not go to school*. *Then*, *after going to school she will keep crying and the teacher tells me not to beat her and let her come to school if she can*.*”*

(Mother of a girl, mobility limitation–“cannot do”)

#### Getting to school

Many children were dropped and picked up from school by their family members for multiple reasons like difficulty in walking, as a safer option (as they got pushed or teased on the way), and for carrying the school bags. They went on a bicycle or motorcycle or walked to school if they could. Therefore, for some children, attending school depended on the availability of a family member to accompany them. Getting children dressed and ready to go to school is an additional challenge for families, particularly those with self-care and cognitive limitations. Difficulty in reaching school because there was no one to accompany the child to school or transport being costly led some parents to discontinue their children from schooling. One of the mothers described the challenge of sending their child to school as:

*“I stopped him because it is difficult to go to school and come back*. *There is no bus in this village*. *We used to go by auto*, *it is Rupees 100 to drop him at school and Rupees 100 in the evening… he studied like this for two years… so we stopped him as we found it difficult*.*”*

(Mother of a boy, mobility limitation–“a lot of difficulty”)

### Experience at school

#### Physical accessibility

Children, particularly those with mobility limitations, faced difficulties independently moving around in the school. For example, some experienced challenges to enter classrooms that were not on the ground floor, going to the playground and toilet or going to the teachers’ or headmasters’ room. This poor physical accessibility restricted some children with disabilities to their classrooms, preventing them from fully participating in all school events, such as school assemblies and social time on the playground during breaks. Requiring children to ask for assistance from their teachers and peers to move around the school could lead to feelings of embarrassment, lack of autonomy, and social exclusion.

*“He stays alone in class when other children are playing*. *I asked why he does not ask his classmates to carry him to the ground and back to class*, *and for that he replied–‘how many days will they take me in and out of class’*. *Not a single child is with him at that time*, *he stays alone in class on the fourth floor*.*”*

(Mother of a boy, mobility limitation–“cannot do”)

The accessibility of school toilets was also a challenge. Several caregivers and children reported that the toilets were generally poorly maintained, with issues such as poor cleanliness, no proper wall around the toilet, and toilets being out of service (e.g., locked, no running water).

Even if on-site toilets were in a usable condition, some children with disabilities faced additional barriers in using them. For example, caregivers of children with cognitive impairments instructed their children to come home if they needed to use the toilet because they are unable to clean themselves. Overall, about a third of children in school–particularly children with mobility and cognitive impairments—returned home to use the toilet. Returning home could lead to time out of school or shortened days. Further, children with physical impairments sometimes needed assistance in using inaccessible on-site toilet facilities. This assistance was often provided by peers, which could lead to feelings of embarrassment and awkwardness:

*“They [peers] are ready to help but my son is not ready to accept their help*. *All his classmates are physically equal to him*, *yet my son fears that they might drop him…*. *children need to lift him*. *The second reason is my son feels embarrassed*, *so we go to school to be with him*.*”*

(Mother of a boy, mobility limitation—“cannot do”)

The mother of a 13-year-old girl with mobility limitations discouraged her daughter from using the school toilet because of a lack of privacy. She said,

*“She goes to toilet only after coming back home after she attained puberty…*. *I tell her not to go in school and tell her to use at home*. *Toilet is not good*. *It is just open without wall… So*, *I tell her to come and use at home*. *She will rarely use in school*.*”*

(Mother of a girl, mobility limitation–“a lot of difficulty”)

The lack of accommodations and poor physical accessibility of schools led in a few instances to families making significant sacrifices to support their child’s schooling. For example, the mother of a boy with a severe physical impairment explained how his father could not go to work because he would accompany their son to school to provide needed assistance:

*“Someone needs to be present in school with him to open the lunchbox and water bottle*, *carry him when he wants to go to bathroom…*., *his father stays there*, *he feeds him food*, *takes him to bathroom*, *and brings him back to home from school*. *My son will say he wants to go to bathroom after having lunch*.*”*

(Mother of a boy, mobility limitation–“cannot do”)

#### Resources for inclusive education

In addition to poor physical accessibility, most schools did not have adequate resources to support inclusive education, particularly for children with sensory and intellectual impairments. For example, many children with visual impairments faced difficulties in reading from the classroom board even when they were seated in the front row. Some had come up with workarounds to gain the required information, such as by asking their peers for their notes:

*“In school*, *anything written on the board will not be visible for me…*. *Only partially it will be visible*, *Sir*. *When classmates write*, *I look at that and write*.*”*

(A boy, visual limitation—“a lot of difficulty”)

Children with profound hearing impairments faced challenges in learning without adapted communication. Some were managing to communicate basic information with friends and teachers with gestures, but communication of more complex concepts such as lessons were challenging without formal sign language. A mother said,

*“She will not read… she writes the words…All the teachers understood that this child cannot hear… they arrange a person to write [her exams] and get her to pass… she is studying in 10*^*th*^, *what is the use*? *She will just go to school… they (teachers) will tell patiently and if she did not understand that they will just leave it*.*”*

(Mother of a girl, hearing limitation–“a lot of difficulty”)

The lack of accommodations could lead to poor learning outcomes for some children. As reported by caregivers, most children were average in their school performance. Still, some caregivers had received complaints from teachers about their children regarding their homework, poor performance in studies, or not being able to complete parts of the lesson (e.g. performing recitations because of a hearing or cognitive impairment), and had advised parents to help the children to learn at home. There was also an indication that class level and marks do not always accurately reflect the actual learning of children with disabilities, particularly children with sensory or intellectual impairments. For example, some children were in secondary school but could not read and write. Further, the use of scribes was reported in seven instances amongst children with sensory and intellectual impairments, in which the school appointed a scribe to write for children who were appearing for board exams so that they would be promoted to the next class:

*“Teachers made someone to write her exam and passed her… she does not know to answer so that they are appointing someone to write for her; she only knows the question and not the answer*.*”*

(Mother of a girl, cognitive limitation—“cannot do”)

*“The headmaster said to stop sending her to school*, *and they will deploy either a child or scribe to write exams for her*…. *she was promoted automatically till 8th standard; they are saying a scribe will write because she is in 10th standard”*

(Father of a girl, cognitive limitation–“a lot of difficulty”)

#### Relationships with peers

The experience of children with their peers at school varied from being helpful to distressing. Caregivers and children alike reported instances of peers playing important roles in social and academic inclusion, such as by carrying their school bag, helping in climbing stairs to reach the classroom, taking them to the toilet, fetching drinking water for them, protecting them from other children who might bully them, and sharing notebooks and helping them to write. Most children in the school reported that spending time with their friends was a major motivator in attending school. Sometimes peers helped them to get back home after school and relayed important information to caregivers.

*“As soon as we leave him at school*, *they [friends] will take his bag and they will keep it in his place… If he asks them to bring some water when he sits and eats*, *they will bring some water and give it to him*. *If he wants to go to any nearby room*, *for example*, *to the headmaster’s room*…*his friends are helping”*

(Mother of a boy, mobility limitation–“a lot of difficulty”)

Still, many children experienced negative interactions with their peers. Over a third of children who had ever been to school had been teased or abused by their peers. Often bullying was disability-targeted, with reports of children being called derogatory names related to their impairments or being picked on because they were perceived to be easier targets (e.g. difficulties running away or reporting bad behaviour to teachers). Children and caregivers reported incidents such as other children stealing their personal items, name-calling, making fun of their poorer school performance, and even being physically beaten.

*“Yes*, *I had questioned them [for calling me names] and fought [with children bullying me]*, *Sir*. *Because my condition is like this*, *they all call like that*, *Sir*. *They frequently tease me*, *Sir*. *Even a single day they do not call me by name*, *Sir*. *My classmates call ‘Jhaadai*, *Jhaadai’[squint-eyed]*.*”*

(A boy, visual limitation–“a lot of difficulty”)

*“She says -I do not know to read*, *for that other children are teasing me… She says—children tease saying that I do not know anything*. *While going to school she will tell me that this boy teased her*, *and that boy teased me*. *Boys*, *and sometimes girls also tease her*.*”*

(Mother of a girl, mobility limitation–“cannot do”)

Even without overt discrimination, some children with disabilities were excluded from social activities at school. Generally, children who could manage to move around independently played with others at school, particularly boys and girls with hearing difficulties. Others, due to the fear of getting hurt, feeling embarrassed to ask for help to go to the playground, or because they might get mocked for losing the game, either were isolated by others or self-isolated, sitting alone in the class or playing indoor games.

*“I don’t like to play because I will fall down… It [playground] is full of gravel I don’t like it*… *I will fall … I will not go to play*. *I will be sitting… They (friends) don’t call me to play because I will fall*.*”*

(A girl, mobility limitation–“cannot do”)

#### Teachers’ attitude

Several teachers were reported to have played positive roles for children with disabilities, such as by providing small accommodations like having children sit in front of the class or giving additional assistance with lessons, and by disciplining classmates who bullied children with disabilities. One of the caregivers said that a teacher living close by had encouraged them to send their daughter with cognitive impairment to school and even accompanied the girl to school.

*“Her teachers look after her kindly*. *She is not able to hear and talk*, *right*, *so they will not hurt her*, *they will teach her and take care of her kindly*.*”*

(Mother of a girl, hearing limitation–“cannot do”)

However, a third of children going to school at the time of the study and half of children who had ever attended school reported verbal abuse or discriminatory behaviour. For example, some teachers were reported to have made derogatory statements about a child’s disability that made the children and the family upset.

*“I do not know whether he did something [wrong] or not*. *Sir [teacher] told him- I will make this eye also blind if you are not obeying… He [son] said that and he cried in the house… Sir [teacher] is telling- I will make another eye blind*.*”*

(Mother of a boy, visual limitation–“a lot of difficulty”)

Further, some teachers discouraged families from sending their child to school, particularly if their child had an intellectual impairment or behavioural challenges. For example, caregivers indicated teachers found it difficult to cope if their child was hyperactive, needed assistance to move around, created disturbances in the classroom, fought with other children, or soiled their clothes. In four instances, teachers reportedly asked caregivers to stop sending their child to school.

*“Her teacher makes her sit away from the other children*. *If they make her sit along with the other children*, *she can study*, *but they make her sit in a corner… The teacher told me not to send her there [school] except when the officials come [for school monitoring visit]*, *for accounting purpose*. *All such problems are there*.*”*

(Aunt of a girl, cognitive limitation–“a lot of difficulty”)

### Learning outside of school

In this setting, it is common for children to receive tutoring outside of school at tuition centres. However, very few children with disabilities (only 4 out of 40) had ever been to tuition centres. Common reasons for not sending children to tuition centres included distance to the centre and tuition being expensive. For example, the mother of a boy with mobility impairment explained that someone would need to take her son to another town, which involved an additional pick up and drop off on top of his school pick up and drop off. Tuition centres were also often not accessible, putting additional stress on children:

*“Yes*, *she was going for tuition in 5th standard; after knee pain*, *we did not send her for tuition*. *Due to frequent knee pain; we cannot send her regularly for tuition*. *She has to climb up [to reach the tuition centre]; she cannot carry her things and climb up*.*”*

(Mother of a girl, mobility limitation–“cannot do”)

Some caregivers and other family members provided support at home, such as checking their child’s homework or helping them understand their studies. For most children, family involvement in their studies was minimal. Still, in a few cases, family members dedicated significant time to helping children learn:

*“We told our daughter to teach for him at night*. *After coming home from college*, *in the evening hours*, *she will teach him for one hour even if she has work to complete…*. *She will teach him English*, *Mathematics and other subjects…*. *when she has more writing work*, *she cannot teach him regularly*.*”*

(Mother of a boy, mobility limitation–“cannot do”)

## Discussion

This qualitative research found that children with disabilities in Tamil Nadu faced multiple barriers to attending school and were often not able to equally partake in learning and social experiences while in school. The study elucidated school “journeys” among children with disabilities, including factors affecting decisions on if and where to enrol, how children got to school, their experience while at school, and learning outside the classroom. This study showed that barriers to enrolling and attending school were present at each of the junctures, including inaccessible facilities, lack of resources for inclusive education, discrimination, and financial difficulties. Children, their caregivers, and teachers were often trying their best to overcome these barriers, with several examples of teachers providing ad hoc accommodations, or caregivers spending significant time and money to support their child’s education. Still, for many children, the persistent institutional and societal barriers led to either non-attendance or limited learning and social inclusion.

Although the school enrolment of children with disabilities is increasing in India, there is a wide variation across states and by gender and type of impairment [19]. For schooling decisions, this, and studies in other areas of India, have highlighted the tension between caregivers recognising the importance of education for their children, but questioning its value when schools did not adequately support their child’s learning, social development, and in some cases safety needs [24, 29]. Concerns over inadequate care and separation of young children from their families are a particular issue for residential special schools, which have been highlighted in other studies [8, 9]. Special schools are often perceived as having more resources to support the learning needs of children with disabilities [30], however, the few children in this study who did attend a special school had poor experiences of care given to the children, leading to dropouts. As such, relying predominantly on special schools is unlikely to advance access to education for children with disabilities. Special schools also do not fulfil the promise of inclusive education that “children that learn together, learn to live together” and is in violation of the UNCRPD [5, 31]. Still, other studies have highlighted the difficulties of adequately resourcing switches from segregated to mainstream schooling, including the availability of specialist teachers and resources [30]. Yet evidence from predominantly high-income settings indicate that learning outcomes tend to be better for children with disabilities in inclusive settings, and inclusive education provides positive social and educational impacts for children without disabilities [32, 33].

Financial barriers were also a dominant challenge to enrolment and regular attendance in both formal and informal schooling. Although education in government schools is free to all in India, poverty is often still a barrier for children with and without disabilities [8]. However, it has been well documented that children with disabilities and their households in India and other settings are more likely to be living in poverty [5, 34]. They also frequently face additional direct or opportunity costs, including attending school [35]. For example, a study in the Philippines found raising a child with a disability required 40–80% more expenditures and households with children with disabilities spent double on education as households without children with disabilities [36]. A common financial barrier to schooling was transport cost in this study and in other studies, particularly for children with mobility limitations or who required supervision to get to school [4, 37, 38]. Further, caregivers often experience high opportunity costs to support their child’s education, taking significant amounts of time to bring their child to and from school, or even to assist them throughout the day due to the lack of accessible facilities and in-school support [29, 39]. Balancing these costs with the need to earn an income could lead to frequent absences or even drop-outs from schools, particularly in poorer families. Social protection and other programmes may need to consider strategies to cover the direct and indirect costs children with disabilities face in attending schools.

While at school, the lack of accessibility and resources for inclusive education affected learning and social inclusion. Inadequate resources for inclusive education for children with disabilities have been noted in other studies in other areas of India and globally [4, 8]. Studies with teachers in India have identified significant challenges to delivering inclusive education, including the lack of training, large class sizes, increased non-teaching responsibilities and inadequate provision of human and material supports [8]. The lack of inclusive education resources carries implications for children’s learning, as there were several examples of children not having skills that would be expected for their grade level or being upgraded to higher grades without passing. Therefore, involvement of specialist educators in mainstream schools would a critical aspect not only to help children with disabilities but to help upskill other teachers [19].

In seven instances, children with sensory or intellectual impairments had received qualifications without taking the exams for themselves. The Rights of Persons with Disabilities Act allows for scribes to support children with disabilities in writing exams under Chapter VII, Section 38(3), but the expectation is that the child is still supposed to answer the questions for themselves [17]. In this study, children with certain disabilities instead of answering the questions themselves, have used the scribes to answer indicating the lack of appropriate educational provision to support the learning of children. Further research is needed to assess how widespread this practice is, as it would have implications on the interpretation of statistics on educational attainment between children with and without disabilities.

This study also highlights the need for inclusive education to go beyond the learning process and consider the social inclusion of children with disabilities in schools. While teachers and peers could be important sources of support and motivators for going to school, in this and other studies, there are also frequent instances of discrimination and abuse [9, 10]. Other Indian studies showed that children are often left out from the friendship groups and playing activities, therefore recommending ‘peer sensitization and empathy building’ as an important aspect of inclusive education [19]. Surveys on teacher attitudes in India have indicated a positive shift in teachers’ attitudes toward children with disabilities and their right to an education in recent years, although teachers also reported feeling overwhelmed and ill-equipped to teach and support children with disabilities [2, 8, 40, 41]. These institutional challenges may partially explain why some caregivers reported that they had been asked to stop sending their children to school–particularly children with high support needs or behavioural difficulties–which is nonetheless a violation of children’s rights under Indian law [16, 17]. Even without overt discrimination, the lack of accessible facilities and other accommodations could lead to social isolation or force children to rely on their peers for often uncomfortable tasks such as using toilets or being carried to and from classrooms. Inaccessible toileting facilities and the lack of support in using these facilities have been highlighted in other studies as a barrier to education for children with disabilities [42, 43]. It is also a particular concern for girls with disabilities as they enter menarche if they cannot manage their menstruation according to social norms [44, 45].

There are several important strengths and limitations to this study that should be taken into account when interpreting the study findings. One limitation was that teachers could not be included in this study, which would be helpful to explore their perspective on delivering education to children with disabilities. Further, some children with severe intellectual impairments and hearing impairments without knowledge of sign language could not be interviewed as they could not communicate with available supports during the study timeframe. These children are arguably most at risk of exclusion, and so further research–potentially involving triangulation with classmates or siblings–is needed to better understand their experiences at school. Still, this study had important strengths. For example, the sample was recruited from a large-scale population-based census, improving the generalisability of results. Qualitative studies with people with disabilities often involve recruitment through civil society organisations, and people affiliated with these organisations may not be reflective of the broader population [46].

Overall, this study carries implications for the delivery of inclusive education and for future research on the access of children with disabilities to education. Importantly, this study emphasises the limitations of traditional quantitative metrics in measuring access to education. Other studies have also found that school attendance, grade level, or attainment may not be indicative of children with disabilities’ learning [47, 48]. Quality of education is a universal challenge affecting all children, but children with disabilities are particularly affected due to the unmet need for inclusive education strategies and supports [49]. As such, indicators for tracking educational progress, including towards meeting the Sustainable Development Goals, may mask the exclusion of children with disabilities from the learning and social experience even if they are in school.

Further research is needed to co-develop and trial innovative strategies to deliver teaching that meets the needs of diverse learners, with input from children with disabilities, caregivers, and teachers on the current gaps and inputs required to overcome them. Overall, there is a lack of evidence on the effectiveness of interventions to improve educational outcomes for children with disabilities–in both India and other settings [50, 51]. India and other settings’ laws and policies reflect a strong commitment to achieving an “inclusive and equitable equality education for all”; however, this commitment is unlikely to be achieved without increased investment and prioritization of disability-inclusive planning in education and other sectors.

## Supporting information

S1 File STROBE statement. (DOC)
